# Outcomes Using Focused Shockwave for Treatment of Bone Stress Injury in Runners

**DOI:** 10.3390/bioengineering10080885

**Published:** 2023-07-25

**Authors:** Alexandra Beling, Amol Saxena, Karsten Hollander, Adam S. Tenforde

**Affiliations:** 1 Spaulding Rehabilitation Hospital, 300 First Avenue, Charlestown, MA 02129, USA; 2 Palo Alto Medical Foundation, Palo Alto, CA 94301, USA; 3 Institute of Interdisciplinary Exercise Science and Sports Medicine, Medical School Hamburg, 20457 Hamburg, Germany

**Keywords:** stress fractures, running, athlete, high energy shock waves

## Abstract

Bone stress injury (BSI) is a common overuse injury that can result in prolonged time away from sport. Limited studies have characterized the use of extracorporeal shockwave therapy (ESWT) for the treatment of BSI. The purpose of this study was to describe the use of ESWT for the management of BSI in runners. A retrospective chart review was performed to identify eligible patients in a single physician’s clinic from 1 August 2018 to 30 September 2022. BSI was identified in 40 runners with 41 injuries (28 females; average age and standard deviation: 30 ± 13 years; average pre-injury training 72 ± 40 km per week). Overall, 63% (*n* = 26) met the criteria for moderate- or high-risk Female or Male Athlete Triad categories. Runners started ESWT at a median of 36 days (IQR 11 to 95 days; range 3 days to 8 years) from BSI diagnosis. On average, each received 5 ± 2 total focused ESWT treatments. Those with acute BSI (ESWT started <3 months from BSI diagnosis) had an average return to run at 12.0 ± 7.5 weeks, while patients with delayed union (>3 months, *n* = 3) or non-union (>6 months, *n* = 9) had longer time for return to running (19.8 ± 14.8 weeks, *p* = 0.032). All runners returned to pain-free running after ESWT except one runner with non-union of grade 4 navicular BSI who opted for surgery. No complications were observed with ESWT. These findings suggest that focused ESWT may be a safe treatment for the management of BSI in runners.

## 1. Introduction

Bone stress injury (BSI) is a common injury in runners and other physically active populations. The injury results from excessive demand on bone, with a spectrum ranging from a stress reaction to stress fracture [1]. When sub-maximal loads placed on a bone exceed its strength, microfractures form. With the progression of loading, a macroscopic fracture line may develop. BSI is a psychologically devastating injury for an athlete because it takes a long time to heal, which includes protracted time away from the sport.

The diagnosis of a BSI is based on the clinical history and physical exam findings, with radiographic imaging to confirm the presence and severity of the injury. Currently, the gold standard for clinical evaluation of BSI is magnetic resonance imaging (MRI). Grading scales using MRI, as originally defined by Fredericson et al., are commonly utilized when assessing the severity of injury: Grade 1 BSI shows periosteal edema on T2 weighted imaging, with normal bone marrow on T1 and T2. Grade 2 BSI shows periosteal and bone marrow edema on T2. Grade 3 BSI shows periosteal edema on T2 with a corresponding dropout on T1. Grade 4 BSI is defined by a visible fracture line, commonly referred to as a stress fracture [2,3,4].

While the MRI grade of BSI has been shown to prognosticate the typical time to return to sport [2,5], other factors may influence the rate of healing [1]. The Female and Male Athlete Triad [6,7] describe the influence of low energy availability on hormonal function and bone health. Low energy availability may contribute to reduced sex hormones (estradiol and testosterone) and lower bone density, resulting in prolonged healing times [2]. The anatomical location of injury can be divided into low-risk and high-risk categories based on the risk of delayed/non-union, complete or re-fracture, or prolonged time for healing [8]. While over 90% of athletes return to sport, some injuries progress to non-union, which can require surgery or result in discontinuation of sport [5]. Surgery with internal fixation, with or without bone grafting, may be effective but has a risk of complications, including failure to achieve union [9].

The initial management of most BSI is non-surgical and typically includes offloading the bone, followed by physical therapy and a gradual return to sport progression. During this time, activities are re-introduced and advanced, provided that they remain pain-free [1]. Identifying methods to promote localized bone healing is desirable for both patients and healthcare providers. Bone stimulators are often considered; however, these have limited evidence for use [10]. While limited to case series, focused extracorporeal shockwave therapy (ESWT) has promising results as a potential method to treat BSI [9,11]. The initial medical use of ESWT was in urology to non-invasively treat kidney stones, termed lithotripsy [12]. Clinical observations of bone hypertrophy on repeat plain radiographs following lithotripsy suggested osteogenic effects on bone [13].

Studies for the use of ESWT in BSI are limited to two case series of stress fractures with non-union. In 2007, Taki reported successful healing of 5 chronic stress fractures in young adult athletes (ages 17–22) in the anterior tibia, medial malleolus, inferior pubic ramus, and base of the fifth metatarsal. One session of high energy focused ESWT (0.29 to 0.4 mJ for 2000 to 4000 shocks) using an electrohydraulic device was performed under spinal anesthesia in an operating room, with radiographic consolidation achieved at 2–3 months and returned to sport 3–6 months following treatment [9]. A subsequent report by Moretti in 2009 documented the use of electromagnetic ESWT at low to moderate energy settings (0.09 to 0.17 mJ) in 10 male soccer players ages 20–29 with stress fractures of the fifth metatarsal or anterior tibia, including a Jones fracture that failed to heal with surgical fixation. Each athlete initially received 3 ESWT treatments for the fifth metatarsal or 4 ESWT treatments to the anterior tibia, with one athlete requiring repeat treatments. Radiographic healing was achieved 6–14 weeks following ESWT, with a successful return to sport in 3–10 months [11]. While these case series document successful management using ESWT, both are limited by small sample sizes and were performed in athletes with non-union of stress fractures. Larger randomized controlled trials have demonstrated healing of traumatic fractures with non-union using ESWT, with success similar to surgical outcomes [14,15,16]. Collective studies to date suggest the potential role of ESWT in the management of bone-related injuries in other populations.

The purpose of this study was to characterize the treatment outcomes of focused ESWT in runners with both acute and chronic BSI. We describe patient factors and characteristics of injury in association with the time of return to pain-free running after ESWT.

## 2. Materials and Methods

### 2.1. Study Procedures

**Figure 1:**
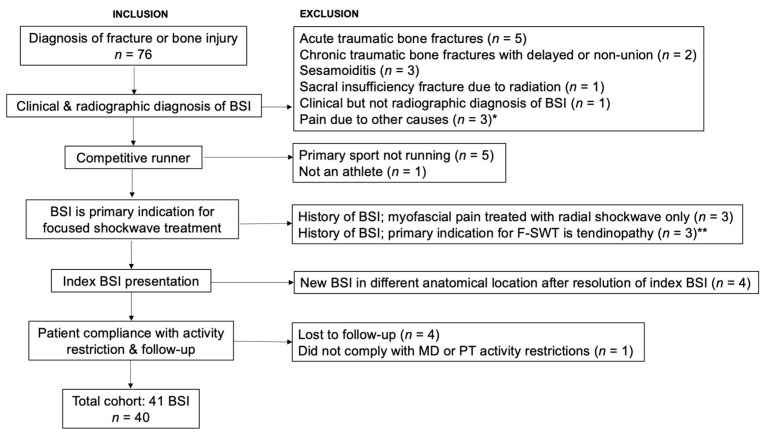
Inclusion and exclusion criteria. * Pain due to other causes: osteoid osteoma (*n* = 1), popliteal artery entrapment (*n* = 1), avascular necrosis of second metatarsal head (*n* = 1) ** History of metatarsal BSI, treated with ESWT for peroneal tendinopathy (*n* = 1); history of tibial BSI, treated for tibialis posterior tendinopathy (*n* = 1); history of calcaneal BSI, treated for concurrent peroneal and tibialis posterior tendinopathy (*n* = 1).

This was a retrospective case series characterizing runners treated for BSI using moderate to high-energy electromagnetic-focused ESWT in a single sports medicine clinic. A full review of billing records identified runners treated with ESWT between 1 August 2018 and 30 September 2022 for management of BSI. Inclusion criteria were: (1) clinical and radiographic diagnosis of BSI; (2) patient’s primary sport was running; (3) focused ESWT was used to treat a primary BSI; and (4) patient compliance with activity restriction and follow-up. Inclusion and exclusion criteria are detailed in Figure 1. In patients who developed a new BSI in a different anatomical location after healing index BSI with ESWT, only the index case was included to avoid response bias. We ultimately identified 40 patients with 41 BSI; one patient had two BSI in separate anatomic locations that were treated concurrently.

### 2.2. Treatment Protocol

Prior to performing ESWT, patients had an initial evaluation, including a history and physical exam, and review of relevant imaging studies. Each patient was screened for Triad risk factors, including low bone mineral density, history of prior BSI or other bone fractures, current or prior eating disorder/disordered eating, menstrual history including age at menarche and secondary oligo- or amenorrhea, or symptoms of low testosterone. Each runner was educated to meet guidelines for calcium and vitamin D intake. Further evaluation and management of Triad risk factors were completed as the standard of care, including additional medical work-up of bone density, i.e., dual-energy X-ray absorptiometry (DXA) and referral to a sports dietician.

The ESWT treatment protocol for each patient was developed by a single sports medicine provider (A.S.T.). As medical insurance does not cover ESWT, a single out-of-pocket fee was charged to perform the full series of ESWT. Patients underwent a minimum of 3 ESWT treatment sessions at the site of BSI using an electromagnetic-focused shockwave device (Duolith, Storz Medical, Tagerwilen, Switzerland). ESWT was performed using anatomical bony landmarks and clinical focusing techniques over sites of maximal pain without the use of anesthesia. Additional radial shockwave was provided to address myofascial components of pain. Clinical follow-up was scheduled on average 6 to 8 weeks later. At that time, physical examination, including single and double leg hop, as well as percussion and palpation of the affected bone, was performed [17,18]. Repeat imaging by X-ray, CT, or MRI was obtained based on the clinical judgment of the sports medicine provider, accounting for anatomical location, severity of injury, and presence of residual pain. For patients with residual pain, additional shockwave treatments were scheduled. For those with no pain on physical exam, return to run was prescribed. Walk-run land progression used intervals of alternating walking and running initially performed every other day, and gradually increased. Alternatively, an anti-gravity treadmill (Alter-G, Fremont, CA, USA) was used, which is an unweighted treadmill that adjusts the percentage of body weight support [19]. Additional ESWT was provided during return to run progression to stimulate further bone consolidation. Every patient was prescribed physical therapy.

### 2.3. Data Processing

BSI grade was classified by MRI on a scale of 1 to 4 using previously established criteria [2,3,4]. Biological risk factors were classified using Female and Male Athlete Triad risk scores [6,7]. The anatomical location of BSI was also categorized as low- or high-risk [1]. Low-risk locations included the posteromedial tibia, fibula, calcaneus, metatarsal head or shaft, sacrum, pubic ramus, or femoral shaft [20]. High-risk locations included the femoral neck, navicular, anterior tibia, medial malleolus, talus, and the base of the 2nd and 5th metatarsals [21]. Delayed union was defined as continued pain at site of BSI, or repeat imaging study with incomplete resolution of injury after 3 months, and non-union determined from initial radiographic diagnosis with ongoing pain after 6 months. For the 3 injuries with delayed union, 1 was confirmed with repeat imaging and 2 by continued pain despite activity restriction (>3 months). For the 9 injuries with non-union, 7 were confirmed by repeat imaging and 2 by the duration of continued pain (>6 months).

### 2.4. Statistics

The primary outcome of interest was the proportion of patients who achieved BSI healing, characterized by time when cleared by a sports medicine physician to begin return to run progression (using anti-gravity treadmill or walk-run progression), and time to return to full body weight land running. Statistical analysis was performed using SPSS software, version 28 (IBM Corp, Armonk, NY, USA). Continuous variables are presented as mean and standard deviation. Categorical variables are described as the proportion of the cohort. Student’s *t*-test was used to compare return to run times between acute and chronic BSI, low and high-grade BSI, and low and high-risk anatomic locations. All tests were two-sided, and *p*-values < 0.05 were considered significant.

## 3. Results

### 3.1. Patient Demographics

The final cohort of 40 patients was predominantly young females with normal BMI, training at moderate weekly mileage volume. Demographic characteristics are summarized in Table 1. Levels of competition varied from high school, collegiate, and recreational to elite runners. Most runners who elected ESWT were referred to the clinic by another provider or were seeking a second opinion (*n* = 28, 70%). Most athletes met the criteria for elevated risk categories based on the Male and Female Athlete Triad cumulative risk assessment. Patients with higher Triad risk scores did not require more shockwave treatments or have a longer time for return to run progression (*p* > 0.05).

### 3.2. Injury Characteristics

**Figure 2:**
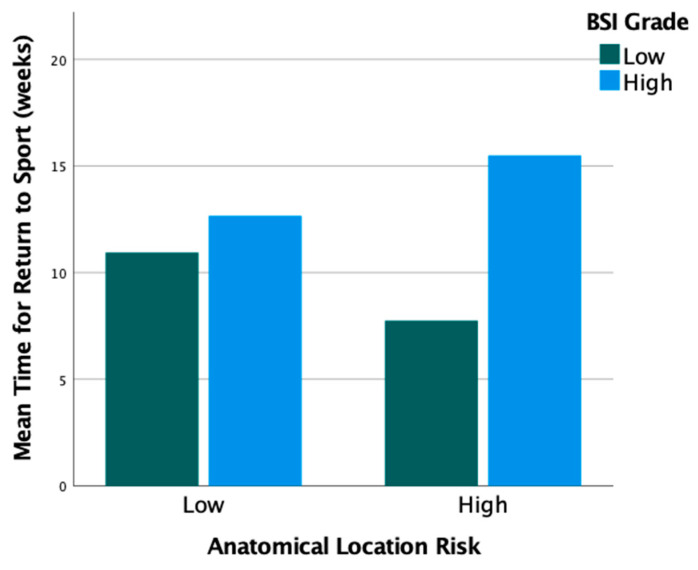
Return to run time (in weeks) by BSI grade and anatomical location risk. Low-risk anatomical locations had an average return to run time of 10.9 ± 9.3 weeks (low grade, *n* = 17) vs. 12.7 ± 9.6 weeks (high grade, *n* = 15, *p* = 0.594). In high-risk anatomical locations, low-grade (*n* = 4) had an average return to run of 7.8 ± 7.1 vs. high-grade (*n* = 5) 15.5 ± 14.4 weeks (*p* = 0.335).

The location of injury was predominantly in the posteromedial tibia or metatarsal. The primary anatomical injury locations are detailed in Table 1. Half of the injuries were low grade using MRI criteria, including grade 1 (*n* = 9, 22%) or grade 2 (*n* = 12, 29%), while the other half were high grade, including grade 3 BSI (*n* = 5, 12%) or grade 4 stress fractures (*n* = 15, 37%). BSI classified using anatomical location was low risk for 32 patients (78%) and high risk for 9 (22%). High-grade BSI in high-risk anatomical locations return to run times were an average of 15.5 ± 14.4 weeks, and low-grade BSI in low-risk anatomic locations had an average return to run time of 10.9 ± 9.3 weeks, although differences did not meet statistical significance (*p* = 0.53) (Figure 2). Further, high-risk anatomical locations did not require more shockwave treatments than low-risk anatomical locations (*p* > 0.05).

### 3.3. Treatment Characteristics

Patients started ESWT at a median of 36 days (IQR 11 to 95 days; range 3 days to 8 years) following BSI diagnosis, and most treatments with ESWT were initiated within 3 months (29 injuries, 72%). However, nearly one-third were treated for delayed healing (diagnosis > 3 months, *n* = 3; 7%) or non-union (diagnosis > 6 months, *n* = 9; 22%). All patients received focused ESWT, while a large portion (*n* = 28, 63%) received concomitant radial shockwave targeting adjacent soft tissue structures using low to moderate energy settings. For focused shockwave treatment, the average energy flux density was 0.37 ± 0.09 mJ/mm^2^, with a minimum of 0.20 mJ and a maximum of 0.55 mJ/mm^2^. Patients received an average of 3122 ± 857 (range 1000 to 5000) pulses per session. An orthopedic surgery consult was obtained in 8 out of 15 stress fractures (53%); of these, 5 (63%) prior to commencing ESWT and 3 (38%) during ESWT.

Patients received an average of 4 ± 1 (range 3–7) focused shockwave treatments prior to return to run, and 1 ± 2 (range 0–10) booster treatments during return to run progression, with an average of 5 ± 2 focused shockwave treatments total.

### 3.4. Radiographic Outcomes

Most runners (*n* = 25, 62%) had imaging after the initial series of ESWT. In 40% (*n* = 16), the imaging was obtained prior to return to run progression, and in 23% (*n* = 9), the imaging was obtained during return to run progression due to concern for re-injury (Table 2).

### 3.5. Clinical Outcomes

**Figure 3:**
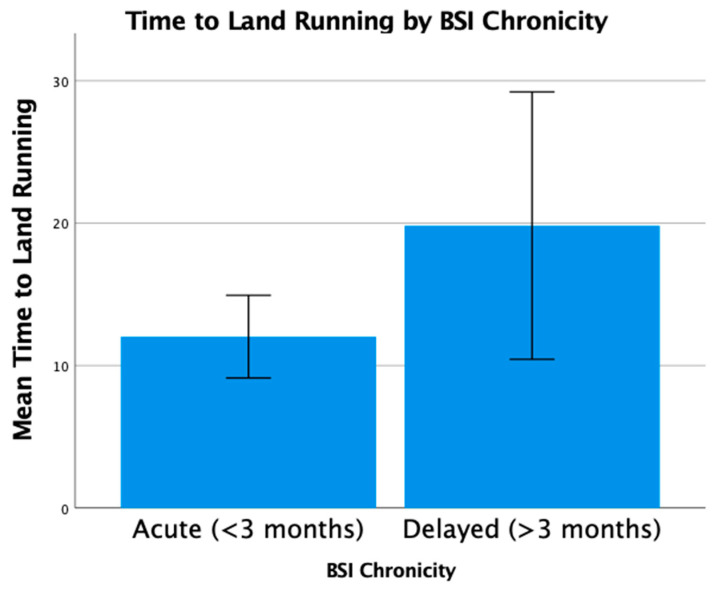
Time to return to land running from beginning shockwave treatment (in weeks) by BSI chronicity. Acute BSI (treatment < 3 months from diagnosis, *n* = 28) had an average return to land running at 12.0 ± 7.5 weeks, while runners with delayed or non-union (*n* = 12) required an average of 19.8 ± 14.8 weeks (*p* = 0.032).

Nearly all runners had successful pain-free return to sport following treatment (*n* = 39, 98%). There was a trend for high-grade BSI (grades 3 and 4) to have longer return to run times than lower-grade injuries (grades 1 and 2), but the difference was not statistically significant (*p* > 0.05). Acute BSI (treatment < 3 months from diagnosis) had an average return to run time of 12.0 ± 7.5 weeks, while runners with delayed or non-union had a longer average return to run time (19.8 ± 14.8 weeks, *p* = 0.032) (Figure 3). Return to run progression was successfully completed using a land-based walk-run progression in most patients (*n* = 25, 63%), with fewer using an anti-gravity treadmill (*n* = 14; 35%). One runner (*n* = 1, 2%) did not have a successful return to running progression with an anti-gravity treadmill. She was an elite runner who had a delayed diagnosis of a navicular stress fracture and failed to achieve pain-free status on return to land running. There were radiographic findings of continued non-union, so she elected for surgical fixation.

### 3.6. Long-Term Outcomes

While all patients reported pain associated with ESWT treatment, there were no unexpected complications with shockwave treatment. Patients were followed for clinical care as indicated for an average of 1 year (minimum 10 weeks, and maximum 3.6 years). Notably, in this cohort, a BSI in a different anatomical location was later diagnosed in 12 runners (30%).

## 4. Discussion

### 4.1. Summary of Findings

The purpose of this study was to describe clinical outcomes following treatment with focused, moderate to high-energy ESWT in a population of runners with BSI. Most runners (*n* = 39, 98%) had resolution of pain and successful return to running, receiving an average of 5 ± 2 total ESWT sessions for management of BSI. As anticipated, a longer return to running time was observed in runners with delayed and non-union BSI. The time for a return to running was similar in low and high-grade BSI using MRI criteria, although a larger sample size may be needed in order to distinguish this difference given the variability in time for the return of sport based on MRI grades and location of injuries [5,8]. Biological risk factors of the Female and Male Athlete Triad did not influence the time for return to run. There was only one athlete with a non-union of a navicular stress fracture who elected for surgery after not achieving a pain-free return to running, which reflects how navicular stress fractures are notoriously difficult to heal [8]. These results are encouraging that most runners were able to return to running without safety complications observed. Side effects associated with shockwave treatment include transient pain and skin erythema. Complications may include local edema or ecchymosis. Across randomized control studies, two patients with Achilles tendon ruptures have been described [22]. There is one case report of humeral head osteonecrosis [23], and another reporting calcaneal stress fracture [24] after ESWT. Notably, surgery has more associated risks than ESWT for the management of fractures [25].

### 4.2. Review of Literature

Shockwave creates compression cycles of negative and positive pressure and high local shear stress in tissue [26,27]. These forces have been shown to cause periosteal detachment and trabecular microfractures with hemorrhage [28,29], thus promoting mesenchymal stem cells into osteoblasts and stimulating osteogenesis [30,31,32]. Furthermore, shockwave liberates endothelial nitric oxide synthase, vascular endothelial growth factor (VEGF), and proliferating cell nuclear antigen to induce angiogenesis and increase blood flow, which promotes healing [33,34].

This report is unique as a large cohort of competitive runners, presenting varying anatomical locations and degrees of BSI. Our treatment protocol is different than what has been previously described by Taki and Moretti [9,11]. Rather than a single session, we used a minimum of 3 moderate to high energy (rather than low to moderate energy) focused shockwave treatments performed once per week to promote BSI healing. Furthermore, no anesthesia was used. This treatment protocol was designed with the goal of promoting bony consolidation by combining prior results from studies using higher energy settings [9] with multiple treatments [11].

A recent meta-analysis encompassing 76 studies and 2974 BSI showed an average return to sport times of 42 days (6 weeks) for Grade 1; 70 days (10 weeks) for Grade 2; 84 days (12 weeks) for Grade 3; and 99 days (14 weeks) for Grade 4 [5]. Taki reported an average return to sport for stress fractures with non-union at 17 weeks [11]. Thus, our return to run times are similar to what has been reported in the literature. Of note, low-grade (grades 1 and 2) BSI had similar return to run times, whether acute or chronic, while in high-grade (grade 3 and 4) BSI, acute high-grade injuries had quicker return to run times than delayed/non-union BSI. Shockwave may be particularly advantageous in healing acute high-grade BSI, with the goal to intervene within 3 months of diagnosis, prior to athletes developing a chronic high-grade BSI (as in the navicular stress fracture case with non-union).

Prior to initiating shockwave treatment, patients were treated with protected weight bearing and loading restriction. No patient was prescribed ultrasound, electromagnetic, electric field, or orthobiologic therapy. While instructed to avoid NSAIDs during and following shockwave therapy, we cannot account for prior non-steroidal anti-inflammatory drugs (NSAIDs) use which may impair and delay the healing of BSI [35,36].

This study is similar to previous studies in the literature in finding a higher prevalence of BSI in females compared to male athletes [37,38]. This is attributed to higher rates of low energy availability in females, including runners [39,40]. Sexual dimorphism may also contribute, with men having larger bones and higher bone mass than females despite comparable body size [41].

We note that 30% of patients sustained a subsequent BSI in a different anatomical location. These findings are consistent with the literature that prior BSI is a strong risk factor for future injury [42,43,44]. The high rate of subsequent injury highlights the challenges of managing BSI in runners, and the importance of a multi-disciplinary treatment approach, including physician, sports dietician, physical therapist, and mental health provider to address the risk factors for impaired bone health and reduce the chance of future injury.

Additionally, we describe the use of “booster treatments” of focused ESWT during return-to-run progression based on the theory that this may promote ongoing and accelerated bone remodeling. Serial imaging of BSI with high-resolution peripheral quantitative CT (HR-pQCT) shows that in the process of remodeling, bone has significantly decreased total, trabecular, and cortical volumetric bone mineral density (vBMD) at 12 weeks from diagnosis, which is the typical time point for when patients are able to return to sport [45,46]. Subsequent HR-pQCT imaging performed at 24 weeks showed that bone density returned to baseline [46]. Thus, during return to run progression, when the newly healed bone is at its most vulnerable, ESWT is used for its osteogenic effect to ensure continued healing, rather than return of injury.

Collectively, these findings suggest that moderate to high-energy focused electromagnetic ESWT is a reasonable and safe treatment option for the management of BSI in runners, particularly in runners with delayed healing or non-union.

### 4.3. Study Strengths and Limitations

The advantages of the present study are the larger sample size than previously presented in the literature and the homogeneity of the treatment protocol, as all patients were treated by a single physician. Limitations of the study are that shockwave is not covered by health insurance and represents an out-of-pocket cost to the patient, which brings in sample bias. However, such data provides a foundation for changing health insurance payor practices. In addition, not all patients had radiographic follow-up, and some clinical information was incomplete by the nature of retrospective chart review (e.g., not all patients had DXA scans). The number of treatments was not standardized and was based on the clinical judgment of the provider and shared decision-making with each patient. We did not include a control group and cannot account for differences in time of healing, particularly for acute BSI. In total, these promising results provide a basis for a randomized controlled trial, which would allow for a control group with sham shockwave, and a comparison of shockwave initiation at more exact time points from BSI diagnosis.

## Figures and Tables

**Table 1:** Demographic and injury characteristics of the study cohort (*n* = 40).

Category	Characteristic	%	*n*
Sex	Female	70%	28
	Male	30%	12
Level of competition	High School	15%	6
	Collegiate	25%	10
	Recreational	48%	19
	Elite	13%	5
Athlete Triad Risk Score	Low	35%	14
	Moderate	48%	19
	High	18%	7
Location	Posteromedial tibia	34%	14
	Metatarsal shaft or head	12%	5
	Metatarsal base	10%	4
	Cuboid	10%	4
	Fibula	10%	4
	Calcaneus	5%	2
	Sacrum	5%	2
	Anterior tibial cortex	5%	2
	Femoral shaft	2%	1
	Lesser trochanter	2%	1
	Inferior pubic ramus	2%	1
	Navicular	2%	1
**Characteristic**	**Units**	**Mean**	**±Standard Deviation**
Age	years	30	± 13
Body mass index (BMI)	kg/m^2^	21	± 2
Pre-injury training volume	km/week	72	± 40

**Table 2:** Summary of follow-up imaging prior to returning to run progression.

Imaging Method	*n*	Mean ± SD (Weeks)	Range (Weeks)	Findings
XR	2	5 ± 1	4–5	persistent sclerosis (*n* = 1) previous fracture line no longer visualized (*n* = 1)
MRI	7	15 ± 7	10–27	improved appearance (*n* = 2) resolution of treated location with new BSI in another location (*n* = 2) resolution of BSI (*n* = 3)
CT	7	10 ± 6	4–22	interval healing (*n* = 2) healed (*n* = 5)

## Data Availability

The data presented in this study are available on request from the corresponding author. The data are not publicly available due to privacy and ethical restrictions.
